# Recent Advances in Mixed-Matrix Membranes for Light Hydrocarbon (C_1_–C_3_) Separation

**DOI:** 10.3390/membranes12020201

**Published:** 2022-02-09

**Authors:** Chong Yang Chuah, Tae-Hyun Bae

**Affiliations:** 1 Department of Chemical Engineering, Universiti Teknologi Petronas, Bandar Seri Iskandar, Perak 32610, Malaysia; 2 CO_2_ Research Centre (CO2RES), Institute of Contaminant Management, Universiti Teknologi Petronas, Bandar Seri Iskandar, Perak 32610, Malaysia; 3 Department of Chemical and Biomolecular Engineering, Korea Advanced Institute of Science and Technology, Daejeon 34141, Korea

**Keywords:** mixed-matrix membrane, zeolite, metal–organic framework, polymers, light hydrocarbon

## Abstract

Light hydrocarbons, obtained through the petroleum refining process, are used in numerous applications. The separation of the various light hydrocarbons is challenging and expensive due to their similar melting and boiling points. Alternative methods have been investigated to supplement cryogenic distillation, which is energy intensive. Membrane technology, on the other hand, can be an attractive alternative in light hydrocarbon separation as a phase change that is known to be energy-intensive is not required during the separation. In this regard, this study focuses on recent advances in mixed-matrix membranes (MMMs) for light hydrocarbon (C_1_–C_3_) separation based on gas permeability and selectivity. Moreover, the future research and development direction of MMMs in light hydrocarbon separation is discussed, considering the low intrinsic gas permeability of polymeric membranes.

## 1. Introduction

Light hydrocarbons (specifically methane, acetylene, ethylene, ethane, propylene, and propane) are major raw materials in petrochemical industries. In particular, the increasing demand for polyethylene, poly(vinyl chloride), ethane, and polypropylene, which are used in the production of everyday materials [1], is driving the substantial production of ethylene and propylene (~191 and 120 million tons, respectively, worldwide in 2019) [2,3,4]. These two hydrocarbons are typically produced through the steam cracking of hydrocarbon sources, such as naphtha, natural gas, coal, and shale gas [5]. Irrespective of the source, the purities of the extracted ethylene and propylene streams should ideally be at least 99.5% [6,7]. The presence of acetylene (>40 ppm) in the ethylene stream, for instance, hampers ethylene polymerization because acetylene could poison the Ziegler–Natta catalyst [8]. This polymerization process is essential because it nullifies contaminants that may trigger side reactions, which could substantially reduce the molecular weight of the resulting polymers [9].

High-pressure cryogenic distillation, a mature technology, has been adopted in industrial operations for light alkene/alkane separation. For example, effective ethylene/ethane separation requires an operating temperature and pressure of −160 °C and 23 bar, respectively, given their similar melting and boiling points (Table 1) [10]; specifically, the boiling and melting points of ethylene and ethane differ by 14 and 15 °C, respectively. Such energy-intensive operating conditions are required for propylene/propane separation as well, because the melting and boiling points of this pair are even more similar (differing by only 6 and 3 °C, respectively). In particular, 75–85% of the input energy is utilized for the effective production of ethylene and propylene [11]. Thus, alternative technologies for effectively separating light hydrocarbons to isolate specific products are highly desirable.

**Figure 1:**
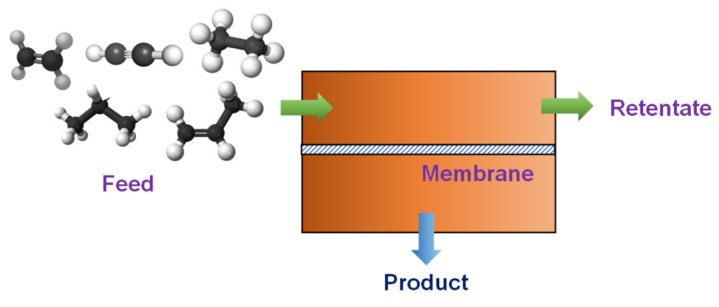
Membranes for gas separation.

Various swing adsorption processes have been proposed to alleviate the high energy penalty in conventional (i.e., pressure- or vacuum-based) processes [10,11,12,13,14,15]. These processes enable hydrocarbon separation at ambient temperature by varying the feed pressure; thus, their energy consumption is relatively low. However, swing adsorption typically entails challenges, such as low recovery, depending on the type of adsorbent used [13,16,17,18,19,20,21]. In addition, their adsorption–desorption cycling is energy-intensive, which restricts their widespread adoption for light hydrocarbon separation [22]. To overcome these drawbacks, membrane-based gas separation technology (Figure 1) has been developed for cost-effective and energy-efficient light hydrocarbon separation [23]. This approach eliminates the need for phase change as separation is achieved under ambient conditions. Polymeric membranes are the main drivers of this technology as they can be fabricated in large sizes with reasonably high mechanical stability [24,25]. However, because the gas transport mechanism is dominated by the solution-diffusion mechanism, the performance of polymeric membrane-based gas separation is governed by the inevitable trade-off relationship between permeability and selectivity [26,27]. Moreover, molecular sieve membranes, developed using pure zeolites and metal-organic frameworks (MOFs), have low scalability due to their poor mechanical strength [28,29,30].

**Table 1:** Properties of light hydrocarbons (C_1_–C_3_) [31,32] .

Light Hydrocarbon	Normal Boiling Point (°C)	Normal Melting Point (°C)	Critical Pressure (MPa)	Critical Temperature (°C)	Kinetic Diameter (Å) ^1^	Van der Waals Diameter (Å) ^1^	Polarizability × 10^25^ (cm^3^)	Dipole Moment × 10^18^ (esu cm)	Quadrupole Moment × 10^26^ (esu cm^2^)
Methane (CH_4_)	−162	−183	4.6	−83	3.80	3.25	25.9	0	0
Acetylene (C_2_H_2_)	−84	−81	6.2	−35	3.30	-	33.3	0	-
Ethylene (C_2_H_4_)	−103	−169	5.1	9	4.16	3.59	42.5	0	1.5
Ethane (C_2_H_6_)	−89	−184	4.9	32	4.44	3.72	44.3	0	0.7
Propylene (C_3_H_6_)	−48	−185	4.6	91	4.68 ^1^	4.03	62.6	0.4	-
Propane (C_3_H_8_)	−42	−188	4.3	97	4.30 ^1^	4.16	62.9	0.1	-

^1^ Hybrid molecular dimension scales based on kinetic diameter and van der Waals diameter are required to obtain a clearer understanding of membrane performance.

Mixed-matrix membranes (MMMs), which combine the advantages of polymeric and molecular sieves (adsorbents), have been a focus of research attention. Numerous papers have reviewed the research on membranes for hydrocarbon separation [2,5,33,34,35]; therefore, the present study focuses mainly on the performance of MMMs and composite membranes in separating C_1_ to C_3_ hydrocarbons. In general, the utilization of MOFs as potential fillers for light hydrocarbon separation has been the main focus due to their higher flexibility in both pore size control and post-synthetic functionalization compared to conventional porous materials, such as zeolites [23]. In addition, their performance with respect to the upper bound limit (C_2_H_4_/C_2_H_6_ and C_3_H_6_/C_3_H_8_) [36,37] is discussed. Finally, insights on the future research and development direction of MMMs in light hydrocarbon separation are shared.

## 2. C_2_ Hydrocarbon Separation

Membranes in C_2_ hydrocarbon separation have been mainly investigated with respect to their potential in C_2_H_4_/C_2_H_6_ separation (Table 2). However, although polymeric membranes, especially those commercially available, are robust and processable, they do not exhibit high permeabilities in ethylene/ethane separation. For example, Naghsh et al. [38], Davoodi et al. [39], and Jeroen et al. [40] demonstrated that the C_2_H_4_ permeabilities of Matrimid^®^, cellulose acetate (CA), and polyimide (P84) under ambient conditions were merely 0.26, 0.051, and 0.049 barrer, respectively, which is attributable to their low fractional free volume (FFV). C_2_H_4_/C_2_H_6_ selectivities, on the other hand, were reported to be moderate, with values of 3.25, 2.27, and 4.00, respectively. It is expected that the reported selectivity is comparatively higher than those of polymers with large FFV, such as 6FDA-based polymers and PIM-1, as observed in CO_2_ separation [24,28,41]. Therefore, these low intrinsic gas permeabilities make the lab-scale (gas chromatography) investigation of such membranes challenging, as it requires specific devices (high-accuracy pressure transducers or thermal conductivity detectors) or conditions (high feed pressure). The advantages of incorporating filler materials into polymeric membranes have been investigated. For instance, Naghsh et al. [38] and Davoodi et al. [39] incorporated amorphous silica nanoparticles into Matrimid^®^ and CA membranes, respectively, and investigated their performance in C_2_H_4_/C_2_H_6_ separation; the incorporation of 20 wt% silica in these two polymeric membranes was found to increase C_2_H_4_ permeability by 58% and 76%, with no substantial change in C_2_H_6_ permeability, in comparison with pure polymer matrix. Although silica nanoparticles are not porous, the residual hydroxyl (–OH) functionalities make interactions with polar C_2_H_4_ molecules feasible. Furthermore, the incorporation of silica nanoparticles disrupts polymer packing, which increases the FFV and facilitates rapid gas transport.

**Figure 2:**
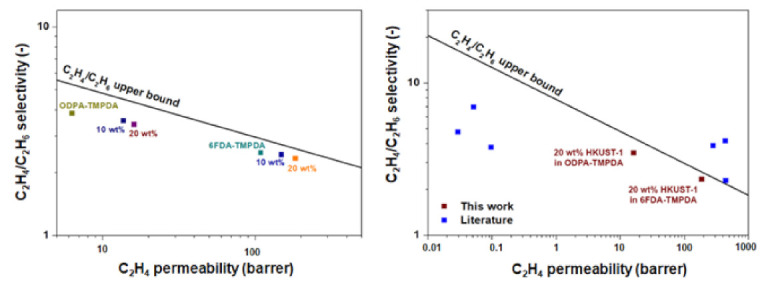
Performance of mixed-matrix membranes (MMMs) with 10 and 20 wt% HKUST-1 loading in ODPA-TMPDA and 6FDA-TMPDA membranes; 20 wt% HKUST-1, whose performance is comparable with those of other membranes reported in previous research, has the potential to surpass the upper bound limit of C_2_H_4_/C_2_H_6_ separation. Reprinted with permission from [44], Creative Commons Attribution 4.0.

These advantages notwithstanding, the use of non-porous silica nanoparticles constrains improvements in C_2_H_4_/C_2_H_6_ separation performance due to their lack of intrinsic porosity, highlighting the need for the incorporation of porous materials into separation membranes. In this regard, MOFs with coordinative unsaturated open metal sites are of particular interest. These metal sites interact favorably with the π-bond in C_2_H_4_, thus facilitating C_2_H_4_ adsorption. Bachman et al. [42] identified M_2_dobdc (M = Co, Fe, Mg, Mn, Ni, Zn), which possess reasonable C_2_H_4_/C_2_H_6_ selectivity (c.a. 8–12 at 50/50 vol/vol C_2_H_4_/C_2_H_6_ and 35 °C, based on ideal adsorbed solution theory [IAST] calculations), as a feasible adsorbent. Studies on MMMs incorporated with M_2_dobdc nanocrystals have revealed that the addition of Mg_2_dobdc and Mn_2_dobdc, unlike Ni_2_dobdc and Co_2_dobdc, creates undesirable interfacial gaps in the polymer matrix, leading to non-selective gas transport [42,43]. Nevertheless, given their high C_2_H_4_ adsorption, these M_2_dobdc-incorporated MMMs have exhibited large increases in C_2_H_4_ permeability, making it increasingly likely to surpass the upper bound limit for C_2_H_4_/C_2_H_6_ separation. The advantages of incorporating MOFs with open metal sites were also demonstrated by Chuah et al. [44], who used HKUST-1 as the adsorbent; specifically, the incorporation of 20 wt% HKUST-1 in 6FDA-TMPDA membranes substantially improved their C_2_H_4_ permeability, pushing their performance close to the C_2_H_4_/C_2_H_6_ upper bound limit (Figure 2).

**Figure 3:**
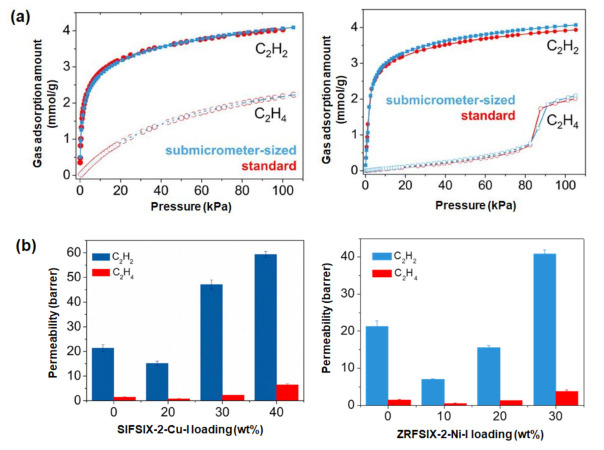
(**a**) C_2_H_2_ and C_2_H_4_ adsorption of SIFSIX-2-Cu-i and ZRFSIX-2-Ni-i at 25 °C; (**b**) C_2_H_2_ and C_2_H_4_ permeability of MMMs with SIFSIX-2-Cu-i and ZRFSIX-2-Ni-i loading in poly(ionic liquids). Reprinted with permission from [45], copyright 2020, Elsevier.

MMMs have also been applied in acetylene separation. However, adsorbents with high acetylene/ethylene selectivity are rare [6]. MOFs are generally more favorable for acetylene/ethylene separation than conventional adsorbents, such as zeolites and activated carbon, because MOFs offer more systematic control over pore size (through the selection of ligands with varying strut lengths) and are compatible with pre- and post-synthetic functionalization. Qian et al. [45] recently demonstrated the C_2_H_2_/C_2_H_4_ selectivity of two types of MOF, namely SIFSIX-2-Cu-i and ZRFSIX-2-Ni-i (Figure 3a); this favorable performance is attributable to the presence of MF_6_^2−^ moieties (M = Cu, Ni), which enables strong interactions with C_2_H_2_. Furthermore, poly(ionic liquids) have also been used in this manner given their reasonable C_2_H_2_ permeability and filler–polymer compatibility [46,47,48]. Specifically, single-gas permeation experiments have shown that 30 wt% SIFSIX-2-Cu-i and ZRFSIX-2-Ni-i in poly(ionic liquids) improve C_2_H_2_ permeability by 129% and 100%, respectively (Figure 3b), without a substantial change in C_2_H_2_/C_2_H_4_ selectivity.

The feasibility of using MMMs for C_2_H_6_/CH_4_ separation has also been explored in research. In contrast to MOFs, which are effective at C_2_H_4_/C_2_H_6_ separation, the applicability of zeolite adsorbents in this separation is relatively less common than in CO_2_ separation (e.g., post-combustion or pre-combustion carbon capture) [49,50] due to the comparable polarizability and quadrupole moment of C_2_H_4_ and C_2_H_6_ molecules (Table 1). Hence, C_2_H_6_/CH_4_ separation was investigated instead due to a more substantial difference in their polarizabilities. Tirouni et al. [51] investigated C_2_H_6_/CH_4_ separation using polyurethane MMMs incorporated with zeolite 4A and ZSM-5. With this zeolite-based approach, the polarizability of C_2_H_6_ is higher than that of CH_4_; accordingly, the permeability of C_2_H_6_ is also higher than that of CH_4_. However, this approach is not promising due to its low C_2_H_6_/CH_4_ selectivity, which in turn is attributable to the poor compatibility between the polymer matrix and the zeolite particles; specifically, the zeolite–polymer configuration has been reported to exhibit the sieve-in-a-cage morphology [52,53,54,55]. These shortcomings can be potentially overcome via post-synthetic treatments, such as the incorporation of silane coupling agents and post-synthetic amine grafting. However, these treatments may jeopardize the effective transport of the desired gas through the membranes [23].

**Table 2:** Performance of mixed-matrix membranes (MMMs) in C_2_ hydrocarbon separation.

Membrane			Measurement Condition		Gas Permeability (Barrer)				Gas Selectivity		Ref.
Polymer/Support	Filler	Loading (wt%)	Pres. (bar)	Temp. (°C)	C_2_H_2_	C_2_H_4_	C_2_H_6_	CH_4_	C_2_H_2_/C_2_H_4_	C_2_H_4_/C_2_H_6_	
6FDA-DAM	Ni-gallate	20	-	-	-	91	25	-	-	4.13	[56]
6FDA- TMPDA	Co2(dobdc)	33	2	35	-	276	71	-	-	3.89	[42]
6FDA- TMPDA	Ni2(dobdc)	25	0.75	35	-	426	101	-	-	4.22	[42]
6FDA- TMPDA	Mg2(dobdc)	23	2	35	-	1140	431	-	-	2.65	[42]
6FDA- TMPDA	Mn2(dobdc)	13	2	35	-	433	188	-	-	2.30	[42]
6FDA- TMPDA	HKUST-1	20	1	35	-	183	76	-	-	2.41	[44]
α-alumina	Zeolite MFI	-	9	25	-	-	37 ^[1]^	6 ^[1]^	-	-	[57]
Cellulose Acetate	Silica	30	-	-	-	0.11	0.026	-	-	4.23	[38]
DBzPBI-BuI	ZIF-8	30	2.7	35	-	112	41	-	-	2.73	[58]
Matrimid	Silica	20	3	30	-	0.42	0.07	-	-	6	[39]
Nylon	Boron Nitride ^[2]^	-	2	-	-	160 ^[1]^	1.9 ^[1]^	-	-	84.2	[29]
ODPA- TMPDA	HKUST-1	20	1	35	-	16	4.7	-	-	3.40	[44]
P1–1 ^[3]^	SIFSIX-2-Cu-i	30	1.5	-	45	2.1	-	-	21.4	-	[45]
P1–1 ^[3]^	ZRFSIX-2-Ni-i	30	1.5	-	15	1.3	-	-	11.5	-	[45]
P84	HKUST-1	20	5	-	-	0.052	0.0075	-	-	6.93	[59]
P84	Fe-BTC	20	5	-	-	0.03	0.0052	-	-	5.77	[40]
P84	MIL-53	20	5	-	-	0.096	0.027	-	-	3.56	[40]
Polystyrene	Fullerene	1	-	35	-	0.58	0.34	-	-	1.71	[60]
Polyurethane	Zeolite 4A	10	2	30	-	-	66.6	53	-	-	[51]
Polyurethane	ZSM-5	20	2	30	-	-	71.3	32.3	-	-	[51]
PPEES	ZIF-8	30	1	-	-	3.2	1.0	-	-	3.2	[61]
PVDF	Ag-Al/NMA	-	0.1	25	-	450	30	-	-	15	[62]

^[1]^ Permeance in GPU; ^[2]^ incorporated with reactive ionic liquid (RIL); ^[3]^ P1-1: (ATMA)(BF4)/PEG_500_ = 1:1 (2-(acryloyloxy)ethyl-trimethylammonium tetrafluoroborate)/poly(ethylene glycol) methyl ether methacrylate; 6FDA = 4,4′-(hexafluoroisoprropylidene)diphthalic anhydride; DAM = 2,4-diaminomesitylene; DBzPBI-BuI = substituted polybenzimidazole; ODPA = 4,4′-oxydiphthalic anhydride; PPEES = poly(1,4-phenylene ether-ether-sulfone); PVDF = polyvinylidene fluoride; TMPDA = 2,4,6-trimethyl-*m*-phenylenediamine.

## 3. C_3_ Hydrocarbon Separation

C_3_ hydrocarbon separation has primarily been examined for the propylene/propane (C_3_H_6_/C_3_H_8_) gas pair (Table 3), with few studies examining the C_3_H_8_/CH_4_ gas pair. As discussed in Section 2, commercial polymer membranes do not exhibit intrinsically high C_3_H_6_ permeability and C_3_H_6_/C_3_H_8_ selectivity due to their considerably larger kinetic diameter compared with other common gaseous molecules, such as CO_2_, N_2_, C_2_H_4_, and C_2_H_6_. Naghsh et al. [38] and Davoodi et al. [39] demonstrated that the C_2_H_6_ permeabilities of Matrimid^®^ and CA under ambient conditions were as low as 0.09 and 0.046 barrer, respectively, due to their low FFV. Thus, it is highly desirable to use polyimide membranes with high intrinsic C_3_H_6_ permeabilities, such as 6FDA-based polymers and rubbery polymers (e.g., polydimethylsiloxane (PDMS]) and cross-linked poly(ethylene oxide) (XLPEO)) to achieve sufficient flux for gas separation.

**Figure 4:**
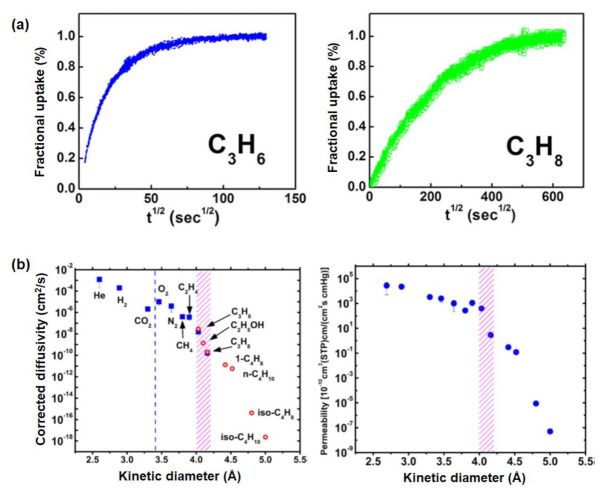
(**a**) Adsorption kinetics of C_3_H_6_ and C_3_H_8_ with ZIF-8 at 35 °C; (**b**) corrected diffusivities and permeability of pure ZIF-8 membrane at 35 °C. Reprinted with permission from [64], copyright 2012, American Chemical Society.

Regarding other porous materials, the effectiveness of ZIF-8 (ZIF = zeolitic imidazolate framework), which is generally stable toward water and organic solvents (e.g., methanol), in C_3_H_6_/C_3_H_8_ separation has been investigated. Based on single-crystal X-ray diffraction data, the aperture of ZIF-8 has been reported to be 3.4 Å [63], which is fairly flexible compared with rigid frameworks (e.g., zeolites and zeotype materials). Thus, considering the flexibility of the ligands present in ZIF-8, Zhang et al. [64] estimated (revised) the effective aperture size. According to the adsorption kinetics of C_3_H_6_ and C_3_H_8_, C_3_H_6_ has a shorter equilibration time than C_3_H_8_ (Figure 4a), indicating a potential means of size discrimination between C_3_H_6_ and C_3_H_8_. However, a precise cut-off for the ZIF-8 aperture size cannot be determined. As an alternative, based on the diffusivities of gaseous molecules against pure ZIF-8 membrane (as calculated from the Maxwell model [23,65]), the diffusion selectivity of C_3_H_6_/C_3_H_8_ has been reported to exceed 100 at 35 °C (Figure 4b); this result indicates the feasibility of using the ZIF-8 aperture size to achieve effective molecular sieving between C_3_H_6_ and C_3_H_8_. As evident from Table 1, kinetic diameter alone is not satisfactory for characterizing the molecular diffusion for selective C_3_H_6_ transport. Thus, as suggested by Zhang et al. [64], a hybrid molecular dimension scale (i.e., a combination of kinetic and van der Waals diameters) is required to effectively explain the feasibility of ZIF-8 to perform effective C_3_H_6_/C_3_H_8_ separation.

Numerous researchers have investigated the use of ZIF-8 for effective C_3_H_6_/C_3_H_8_ separation. The first widely known investigation of ZIF-8 as a filler in MMMs for C_3_H_6_/C_3_H_8_ separation was conducted by Zhang et al. [66]. Based on the gas adsorption isotherms and adsorption kinetics of both C_3_H_6_ and C_3_H_8_, their sorption capacities and fractional uptake are comparable. The incorporation of 48 wt% ZIF-8 in 6FDA-TMPDA membranes was found to increase the C_3_H_6_ permeability and C_3_H_6_/C_3_H_8_ selectivity by 258% and 150%, respectively. These conflicting results are attributable to the difficulty of accurately characterizing the adsorption kinetics (i.e., determining the mass and heat transfer resistance and particle size) during the equilibrium gas adsorption measurements [12,67].

**Figure 5:**
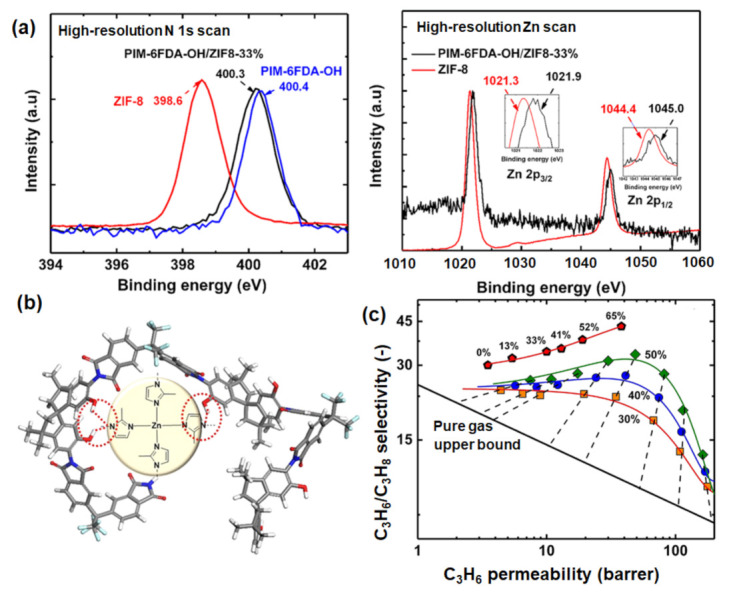
(**a**) Effect of incorporating ZIF-8 in PIM-6FDA-OH on the Zn and N signals in XPS spectra. (**b**) Illustration of the proposed mechanism, based on hydrogen bonding between ZIF-8 and the polymer (PIM-6FDA-OH); (**c**) C_3_H_6_/C_3_H_8_ separation performance with varying ZIF-8 loading. Theoretical prediction at 30 wt% (orange), 40 wt% (blue), and 50 wt% (green) ZIF-8 are illustrated for comparison. Reprinted with permission from [69], copyright 2018, American Chemical Society.

Efforts have also been made to improve the separation performance of ZIF-8-containing MMMs. Specifically, polymers other than 6FDA-TMPDA [68] have been investigated with the aim of surpassing the upper bound limit for C_3_H_6_/C_3_H_8_ separation. For example, Ma et al. [69] examined the use of PIM-6FDA-OH, which possesses higher C_3_H_6_/C_3_H_8_ selectivity (30 vs 12 for 6FDA-TMPDA) due to the hydroxyl (–OH) group. As evidenced by X-ray photon spectroscopy (XPS) data (Figure 5a), hydrogen bonding (N…H–O) between the nitrogen moiety in the ZIF-8 ligand and the –OH group in the polymer improves the compatibility between the filler and the polymer (Figure 5b), in turn improving the C_3_H_6_ permeability and C_3_H_6_/C_3_H_8_ selectivity by 1.086% and 43%, respectively (Figure 5c), at 65 wt% ZIF-8. The incorporation of ZIF-8 in rubbery polymers, such as PDMS and XLPEO, has also been explored given their high C_3_H_6_ permeability [70,71], but these compounds have yet to be used in industrial operations due to their low inherent brittleness [24].

**Figure 6:**
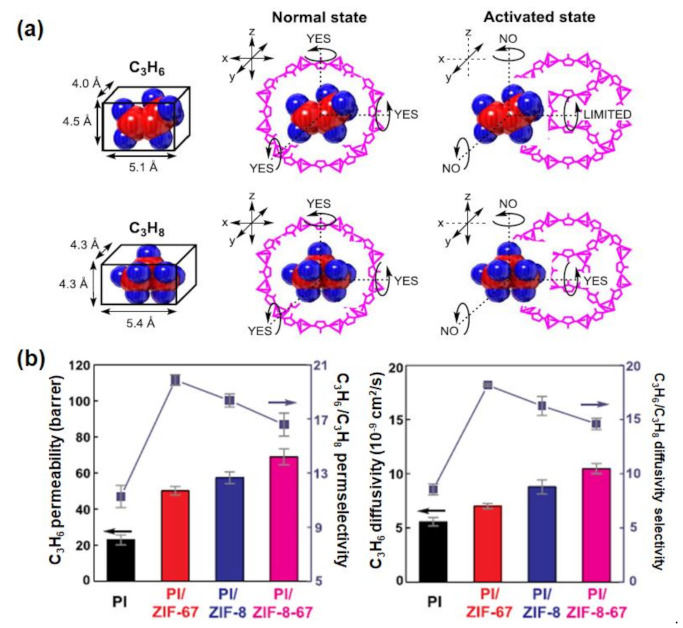
(**a**) Illustration of the effect of transport of C_3_H_6_ and C_3_H_8_ upon passing through the pores of ZIF-67. Reprinted with permission from [75], copyright 2017, Elsevier; (**b**) effect of incorporating ZIF-67, ZIF-8, and ZIF-8-67 at 21.9 wt% loading in polyimide (PI) on C_3_H_6_ permeability, C_3_H_6_/C_3_H_8_ selectivity, C_3_H_6_ diffusivity, and C_3_H_6_/C_3_H_8_ selectivity. Reprinted with permission from [76], copyright 2020 Elsevier.

Other porous materials have also been explored for C_3_H_6_/C_3_H_8_ separation. For instance, cobalt-substituted ZIF-8 (ZIF-67) has been proposed to be more efficient in this regard than ZIF-8 because the Co–N bond in ZIF-67 is stiffer than the Zn–N bond in ZIF-8. In particular, due to the higher electronegativity of Co compared with Zn, the generation of a stiffer ionic bond has the potential to restrict the flipping motion of the organic ligands present in the respective MOFs (Figure 6a) [72,73,74]. An et al. [75] demonstrated that the incorporation of 20 wt% ZIF-67 in 6FDA-TMPDA improved the C_3_H_6_/C_3_H_8_ selectivity by 165%, whereas a comparable ZIF-8 incorporation resulted in an 83% improvement. Furthermore, Oh et al. [76] developed Co^2+^ and Zn^2+^ mixed-metal hybrids (ZIF-8-67) to take advantage of the benefits of both fillers. A comparison study of ZIF-8, ZIF-67, and ZIF-8-67 revealed that ZIF-8-67 could improve C_3_H_6_ permeability to a much larger extent than could the ZIF-8 and ZIF-67 frameworks (Figure 6b). The use of composite fillers, such as ZIF-67 with porous graphene oxide (GO) [77], has also been explored. The creation of three-dimensional architectures with MOFs and GO has been reported to increase the accessible surface area due to the favorable interactions between the functional groups in GO and the ligands in MOFs [78,79,80].

Frameworks other than those involving ZIFs have also been investigated in previous research. For example, Liu et al. [81] examined the potential of Zr-*fum*-fcu-MOF crystals in C_3_H_6_/C_3_H_8_ separation and found that the resulting C_3_H_6_/C_3_H_8_ sorption selectivity is comparable with that of ZIF-8, and a pore aperture size of 3.3–4.6 Å was found to further improve the C_3_H_6_/C_3_H_8_ diffusion selectivity. Furthermore, it has been postulated that C_3_H_8_ has a comparatively higher energy barrier to pass through Zr-*fum*-fcu-MOF crystals than does C_3_H_6_, due to the C–C bond rotation of C_3_H_8_. Similarly, Lee et al. [82] developed defect-engineered MOFs derived from hydrolytically-stable [83] UiO-66 to create additional sorption sites for effective C_3_H_6_/C_3_H_8_ separation; their defect-engineered UiO-66-based MMM significantly improved C_3_H_6_ permeance (1.005%) relative to the control (UiO-66-based MMM, 308%), without substantially jeopardizing the C_3_H_6_/C_3_H_8_ selectivity.

**Table 3:** Performance of mixed-matrix membranes (MMMs) in C_3_ hydrocarbon separation.

Membrane			Measurement Condition		Gas Permeability (barrer)			Gas Selectivity	Ref.
Polymer/ Support	Filler	Loading (wt%)	Pres. (bar)	Temp. (°C)	C_3_H_6_	C_3_H_8_	CH_4_	C_3_H_6_/C_3_H_8_	
6FDA- Durene	ZIF-8	30	2	35	49	2.8	-	17.5	[71]
6FDA- TMPDA	UiO-66	20	2	35	87.4	8.9	-	9.82	[82]
6FDA- TMPDA	UIO-TF36	20	2	35	236.5	24.5	-	9.65	[82]
6FDA- TMPDA	ZIF-67	20	2	35	47.8	3.7	-	14.06	[77]
6FDA- TMPDA	ZIF-67	20	-	35	34.1	1.1	-	31.00	[75]
6FDA- TMPPDA	ZIF-67/GO (ZGO67)	20	2	35	43.1	3.1	-	13.90	[77]
6FDA- TMPDA	ZIF-8	20	-	35	27.7	1.8	-	15.39	[75]
6FDA- TMPDA	ZIF-8	48	2	35	52	2.4	-	21.67	[69]
6FDA- TMPDA	ZIF-8	48	2	35	56.2	1.8	-	31.22	[66]
6FDA- TMPDA	ZIF-8	30	2	35	15	1	21	15.00	[68]
6FDA- TMPDA	ZIF-8	30	2	35	10 ^[1]^	1.5 ^[1]^	-	6.67	[68]
6FDA- TMPDA	ZIF-8	30	2	35	35	1.7	-	20.59	[71]
6FDA- TMPDA	ZIF-8–67	21.9	2	35	65	3.9	-	16.67	[76]
6FDA- TMPDA	ZPGO67	20	2	35	55.4	3.9	-	14.21	[77]
6FDA- TMPDA	Zr-fum-fcu-MOF	15	1	35	40.4	2.8	-	14.43	[81]
6FDA- TMPDA (with PDMS)	ZIF-8	30	2	35	6 ^[1]^	0.4 ^[1]^	-	15.00	[68]
α-alumina	Zeolite MFI	-	9	25	-	300	6 ^[2]^	-	[57]
Cellulose Acetate	silica	30	-	-	0.092	0.018	-	5.11	[38]
Comb copolymers	AgBF4/MgO nanosheet (3:7)	-	-	-	11.8 ^[1]^	0.9 ^[1]^	-	13.11	[84]
DBzPBI-BuI	ZIF-8	30	2.7	35	11	0.4	-	27.50	[58]
Ethyl Cellulose	Fullerene	10	1	-	10	3	-	3.33	[85]
Matrimid	Silica	20	3	30	0.16	0.008	-	20.00	[39]
PEBAX^®^ 1657	ZIF-8	30	2	35	195	78	-	2.50	[71]
PEBAX^®^ 2533	ZIF-8	20	2	35	420	220	-	1.91	[71]
PDMS	ACN-N	10	4	35	-	11,000	1000	-	[86]
PDMS	ACN-O	10	4	35	-	13,000	1050	-	[86]
PDMS	SiO2	10	5	35	9800	-	-	-	[87]
PDMS	ZIF-8	20	2	-	-	160 ^[1]^	-	-	[70]
PIM- 6FDA-OH	ZIF-8	65	2	35	35	1.1	-	31.81	[69]
Polyurethane	Zeolite 4A	10	2	30	-	78	53	-	[51]
Polyurethane	ZSM-5	20	2	30	-	71.3	32.3	-	[51]
PVAc	ZIF-8	40	2	35	24	2	-	12	[71]
XLPEO	ZIF-8	42	2	35	28	1.9	-	14.73	[71]
XLPEO	ZIF-8-IT	20	-	35	11.6	1.8	-	6.44	[88]
XLPEO	ZIF-8-NC	20	-	35	13.8	1.9	-	7.26	[88]
XLPEO	ZIF-8-NR	20	-	35	16.6	1.8	-	9.22	[88]
XLPEO	ZIF-8-OP	20	-	35	12.5	2.2	-	5.68	[88]
XLPEO	ZIF-8-RD	20	-	35	10.2	1.9	-	5.37	[88]

^[1]^ Permeance in GPU; 6FDA = 4,4′-(hexafluoroisoprropylidene)diphthalic anhydride; CAN = adsorptive carbon nanoparticles; DBzPBI-BuI = substituted polybenzimidazole; IT = interpenetration twin; NC = nanocube; NR = nanorod; OP = octagonal plate; PIM = polymer of intrinsic microporosity; PDMS = polydimethylsiloxane; PVAc = polyvinyl acetate; TF = trifluoroacetic acid; TMPDA = 2,4,6-trimethyle-m-phenylenediamine; XLPEO = crosslinked poly(ethylene oxide).

## 4. Comparison of Membrane Performance against the Upper Bound

**Figure 7:**
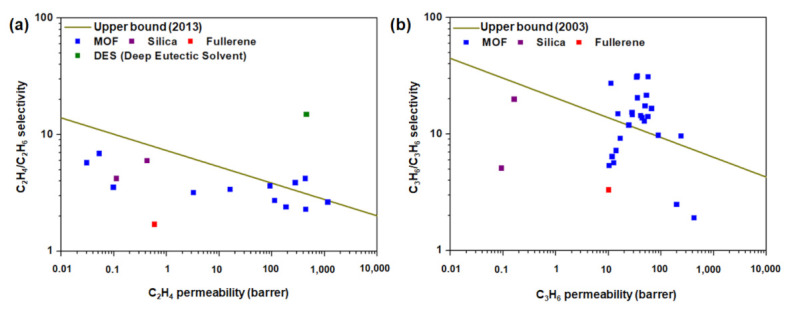
Performance of MMMs in (**a**) C_2_H_4_/C_2_H_6_ and (**b**) C_3_H_6_/C_3_H_8_ separation. The data points indicated in Panels (**a**) and (**b**) are from Table 2 and Table 3, respectively. The parameters used to generate the upper bound line [36,37] are summarized in Table 4.

In this study, the hydrocarbon separation performance of membranes is benchmarked against the upper bound curve for C_2_H_4_/C_2_H_6_ and C_3_H_6_/C_3_H_8_ separation, based on the data in Table 2 and Table 3, respectively. The parameters used to obtain the upper bound plot (Figure 7) are listed in Table 4. The results reveal that achieving excellent light hydrocarbon separation performance is highly challenging. For example, the trend in Figure 7a indicates that the upper bound limit could easily be surpassed by using polymeric membranes with M_2_dobdc series fillers, considering their high C_2_H_4_ adsorption and C_2_H_4_/C_2_H_6_ selectivity (based on IAST calculations). However, MOFs with open metal sites generally exhibit poor hydrolytic stability, which may affect their long-term separation performance. The incorporation of deep eutectic solvents (DES) into polymeric membrane [62] has been proven to be feasible in substantially improving C_2_H_4_/C_2_H_6_ selectivity, the challenges of developing cost-effective and highly stable DES notwithstanding [89,90]. As discussed in Section 3, the use of ZIF-8 as porous material in membranes for C_3_H_6_/C_3_H_8_ separation has been proven effective, as ZIF-8 substantially improves C_3_H_6_/C_3_H_8_ selectivity. Thus, the incorporation of ZIF-8 in polymeric membranes has the potential to surpass the C_3_H_6_/C_3_H_8_ upper bound limit, as indicated in Figure 7b.

## 5. Conclusions and Future Perspectives

This perspective paper presents an overview of MMMs used for C_2_H_4_/C_2_H_6_ and C_3_H_6_/C_3_H_8_ separation. Such light hydrocarbon separations are generally challenging due to the similar polarizabilities, kinetic diameters, and van der Waals diameters of the adsorbates, which make it difficult to realize high selectivity. Moreover, because light hydrocarbon molecules are bulkier than carbon dioxide, nitrogen, and methane (which have been extensively investigated for CO_2_ separation [23,28,91]), membranes with high FFV are highly desirable for separation. Thus, most relevant studies have investigated high-performance polyimides, namely 6FDA-based polymers and PIMs [44,92,93]. However, these polymers require extensive monomer purification, making their large-scale fabrication difficult [53]. Composite membranes are thus a feasible alternative for light hydrocarbon separation. This can be potentially achieved with the utilization of porous fillers possessing large accessible surface areas that are not limited to zeolites and MOFs, as they can effectively increase the diffusion rates of gas-molecule MMMs.

Future efforts should focus on the investigation of MMMs for acetylene separation. Acetylene is typically produced via the thermal cracking of CH_4_ and typically contains CO_2_ as the byproduct. Acetylene is also present as an impurity in C_2_H_4_ production, which tends to disrupt the effective polymerization of C_2_H_4_. Acetylene separation is largely reliant on the use of adsorbents, but effective membrane-based separation is difficult because of the comparable properties of the adsorbates (Table 1). Nevertheless, the membrane performance of MMMs can be expected to improve through the use of porous materials proven to be effective in previous studies.

## Figures and Tables

**Table 4:** Parameters used to obtain the upper bound for C_2_H_4_/C_2_H_6_ and C_3_H_6_/C_3_H_8_ separation.

Upper Bound Curve	2003 (2013) ^[1]^	
	*λ* ^[2]^	*β* ^[2]^
C_2_H_4_/C_2_H_6_	0.14	7.3
C_3_H_6_/C_3_H_8_	0.17	20.4

^[1]^ The upper bounds for C_2_H_4_/C_2_H_6_ and C_3_H_6_/C_3_H_8_ are based on the data obtained in 2013 and 2003, respectively; ^[2]^ *λ* and *β* are described as the slope and front factor of the upper bound line, for which the relation between permeability and selectivity can be correlated as follows: $\alpha =\left(\beta /P^{\lambda }\right)$.

## Data Availability

Not applicable.
